# Efficacy of arthrocentesis and PRP in TMJ osteoarthritis patients

**DOI:** 10.6026/973206300221004

**Published:** 2026-02-28

**Authors:** Cheena Singh, Ashish Kalawat, Kiran Kumari, Abhigyan Manas, Madhuri S. Sale, Pushkar Gupta, Rajat Misurya

**Affiliations:** 1 Department of Oral Medicine and Radiology, Post Graduate Institute of Dental Sciences, Rohtak, Haryana, India; 2 Department of Orthodontics and Dentofacial Orthopaedics, Maharaja Ganga Singh Dental College and Research Centre, Sri Ganganagar, Rajasthan, India; 3 Department of Dentistry, Autonomous State Medical College, Firozabad, Uttar Pradesh, India; 4 Department of Oral and Maxillofacial Surgery, Faculty of Dental Sciences, Uttar Pradesh University of Medical Sciences, Saifai, Etawah, Uttar Pradesh, India; 5 Department of Oral Pathology and Microbiology, Bharati Vidyapeeth (Deemed to be University) Dental College and Hospital, Sangli, Maharashtra, India; 6 Department of Prosthodontics and Crown & Bridge, Hitkarini Dental College and Hospital, Jabalpur, Madhya Pradesh, India; 7 Department of Dentistry, Maharani Laxmi Bai Medical College, Jhansi, Uttar Pradesh, India;

**Keywords:** Arthrocentesis, mandibular function, platelet-rich plasma (PRP), pain management, temporomandibular joint osteoarthritis

## Abstract

Arthrocentesis has emerged as a well-established minimally invasive procedure for temporomandibular joint osteoarthritis (TMJ-OA). Conservative therapies often provide limited and short-term symptomatic relief. Therefore it is of interest to assess the clinical efficacy of arthrocentesis combined with platelet-rich plasma (PRP) injection in reducing pain and recovering mandibular function in patients with TMJ osteoarthritis. Hence, total 38 patients diagnosed with TMJ-OA underwent arthrocentesis followed by intra-articular injection of autologous PRP. Clinical outcomes assessed included pain intensity using the Visual Analog Scale (VAS) and maximum mouth opening at 1, 3 and 6 months. Arthrocentesis combined with PRP provides sustained pain relief and functional improvement in TMJ-OA.

## Background:

Temporomandibular joint osteoarthritis (TMJ-OA) is a progressive degenerative disorder characterized by cartilage deterioration, subchondral bone remodeling, synovial inflammation and subsequent impairment of joint function. Clinically, it presents with chronic pain, joint sounds, restricted mandibular movements and compromised quality of life. Conventional management strategies, including occlusal splints, pharmacotherapy and physiotherapy, often provide only temporary symptomatic relief, particularly in moderate to advanced stages of the disease, thereby necessitating minimally invasive interventional approaches [1]. Arthrocentesis has come out as a well-established minimally invasive procedure for TMJ-OA, primarily aimed at lavage of the superior joint space to remove inflammatory mediators, release intra-articular adhesions and restore joint mobility. Numerous clinical trials have demonstrated that arthrocentesis effectively reduces pain and improves mouth opening; however, its therapeutic benefits are largely mechanical and anti-inflammatory, with limited potential for biological regeneration of degenerated joint tissues. Consequently, adjunctive intra-articular therapies have been explored to enhance and prolong treatment outcomes. Among these, platelet-rich plasma (PRP) has gained increasing attention due to its autologous origin and regenerative potential [1]. PRP is a concentrated preparation of platelets containing a high level of growth factors such as transforming growth factor-β, platelet-derived growth factor and vascular endothelial growth factor, which play a crucial role in modulating inflammation, promoting angiogenesis and facilitating tissue repair.

Randomized clinical trials have reported superior pain decrease and functional improvement when PRP is used as an adjunct to arthrocentesis compared with hyaluronic acid or arthrocentesis alone, suggesting a synergistic effect between mechanical lavage and biological modulation of the joint environment [1, 2]. Recent evidence further supports the clinical efficacy of PRP either as a standalone intra-articular injection or in combination with arthrocentesis for the management of TMJ-OA. Single-blinded and randomized controlled trials have demonstrated significant improvements in pain scores, mandibular function and joint sounds with combined treatment protocols [3, 4-5]. Meta-analyses and Systematic reviews have reinforced these findings, indicating that PRP-based interventions offer superior and longer-lasting outcomes compared to conventional intra- articular agents, although heterogeneity in preparation protocols remains a concern [6]. Despite growing evidence, the optimal role of PRP as an adjunct to arthrocentesis in TMJ-OA continues to be investigated. Recent systematic reviews of randomized controlled trials have emphasized the need for well-designed clinical studies to further validate the efficacy, safety and clinical predictability of orthobiologic agents in TMJ-OA management [7]. Therefore, it is of interest to assess the clinical efficacy of arthrocentesis combined with platelet-rich plasma injection in patients with temporomandibular joint osteoarthritis, with a focus on pain reduction and functional improvement over a defined follow-up period.

## Materials and Methods:

This prospective interventional research was done on 38 patients diagnosed with temporomandibular joint osteoarthritis based on clinical findings and radiographic evidence. The sample size was calculated using the formula n = Z^2^σ^2^ / d^2^, where Z represented the standard normal variate at a 95% confidence level (1.96), σdenoted the estimated standard deviation from previous literature and d indicated the allowable error; the calculated sample size was rounded to 38 subjects. Patients aged 1860 years with persistent TMJ pain restricted mouth opening and failure to respond to conservative management were included, while those with systemic inflammatory arthritis, prior TMJ surgery, bleeding disorders or active infection were excluded. All participants underwent arthrocentesis of the affected TMJ using a standard two-needle technique under aseptic conditions, followed by intra-articular injection of autologous platelet-rich plasma prepared by a double-spin centrifugation method. Clinical parameters including pain intensity (Visual Analog Scale), maximum mouth opening, joint sounds and mandibular function were recorded preoperatively and at scheduled follow-up intervals. The collected data were evaluated using a relevant descriptive and inferential statistical tests, with a p-value of <0.05 considered statistically considerable.

## Results:

All 38 patients completed the research and were included in the final analysis. The study population demonstrated a marked improvement in clinical outcomes following arthrocentesis combined with platelet-rich plasma (PRP) injection. Pain intensity and functional parameters showed statistically significant changes when preoperative values were compared with postoperative follow-up assessments. A statistically considerable decrease in pain scores was observed at all postoperative intervals compared to baseline. Repeated measures analysis demonstrated a progressive decline in VAS scores over time, indicating sustained pain relief following arthrocentesis with PRP injection (p< 0.001) (Table 1). A considerable improvement in maximum mouth opening was noted at the 6-month follow-up, with a mean increase of 12.2 mm (p< 0.001). Additionally, the prevalence of joint sounds and mandibular functional limitation scores showed a statistically significant reduction, reflecting enhanced joint function and reduced mechanical symptoms after treatment (Table 2). The combination of arthrocentesis and PRP injection resulted in significant improvement in both subjective and objective clinical parameters among patients with TMJ osteoarthritis. Pain reduction was rapid and sustained, while functional outcomes such as mouth opening and mandibular mobility improved progressively over the follow-up period. Statistical analysis confirmed that these improvements were highly significant, supporting the efficacy of arthrocentesis with PRP as an effective minimally invasive therapeutic modality for TMJ osteoarthritis management.

## Discussion:

TMJ-OA multifactorial degenerative pathological changes are driven by elevated intra-articular concentrations of inflammatory cytokines, proteolytic enzymes and oxidative stress mediators, resulting in persistent pain, joint sounds and restricted mandibular movements. Conventional conservative therapies often fail to address the intra-articular inflammatory milieu, necessitating minimally invasive interventions that target both mechanical and biological components of the disease process [1, 2-3]. In the present study, arthrocentesis combined with platelet-rich plasma (PRP) injection produced statistically considerable and clinically consequential improvements in both pain intensity and functional parameters. All 38 patients demonstrated progressive symptom resolution over the follow-up period, underscoring the therapeutic efficacy and clinical predictability of this combined approach. The significant reduction in Visual Analog Scale (VAS) scores-from a preoperative mean of 7.24 ± 1.02 to 1.96 ± 0.73 at 6 months-reflects not only rapid analgesia but also sustained pain control. This progressive decline suggests that the treatment effect was not transient, but rather cumulative and biologically mediated.The early reduction in pain observed at the 1-month follow-up (mean VAS reduction of 3.06; p< 0.001) can be primarily attributed to the mechanical lavage effect of arthrocentesis. Lavage reduces intra-articular pressure, eliminates inflammatory mediators such as prostaglandins and interleukins and releases fibrous adhesions, thereby interrupting the pain cycle. Similar early pain relief following arthrocentesis has been consistently reported in randomized clinical trials [1, 5]. However, the continued and significant pain reduction noted at 3 and 6 months postoperatively suggests an additional biological mechanism beyond simple lavage. The adjunctive use of PRP likely played a pivotal role in sustaining and enhancing pain relief. PRP contains high concentrations of growth factors, including platelet-derived growth factor and transforming growth factor-β, which modulate inflammatory pathways and promote tissue repair. Randomized controlled trials have demonstrated superior long-term pain reduction when PRP is combined with arthrocentesis compared to arthrocentesis alone or arthrocentesis with hyaluronic acid [1, 2]. The findings of the present study are in close agreement with these reports, reinforcing the hypothesis that PRP provides prolonged anti-inflammatory and regenerative effects within the TMJ environment. Functional outcomes in this study further support the synergistic efficacy of arthrocentesis and PRP. Maximum mouth opening increased significantly from a preoperative mean of 29.6 ± 4.1 mm to 41.8 ± 3.6 mm at 6 months, representing a mean gain of 12.2 mm (p< 0.001). This improvement is clinically significant and reflects restoration of joint mobility and reduction in mechanical obstruction. Arthrocentesis contributes to immediate improvement by lysing adhesions and restoring joint lubrication, while PRP enhances synovial health and cartilage metabolism, facilitating long-term functional recovery. Comparable improvements in mandibular mobility have been reported in previous randomized trials and systematic reviews evaluating PRP-augmented arthrocentesis protocols [3, 5-6]. The marked reduction in joint sounds-from 71.1% preoperatively to 21.1% at 6 months-further indicates improved joint mechanics and disc- condyle coordination. Joint sounds in TMJ-OA are often associated with surface irregularities, disc displacement and synovial inflammation. The observed reduction suggests improved articular surface smoothness and reduced inflammatory friction, consistent with the biological effects of PRP on cartilage and synovial tissue. Similar reductions in joint sounds have been documented in studies comparing combined arthrocentesis-PRP therapy with standalone treatments [3, 5]. Mandibular function limitation scores also showed a significant decrease, reflecting improved masticatory efficiency and patient- perceived functional ability. This multidimensional improvement-encompassing pain, mobility and functional performance-highlights the comprehensive therapeutic benefit of combining mechanical and biologic interventions. Meta-analyses and systematic reviews have corroborated these findings, concluding that PRP-based intra-articular therapies offer superior functional outcomes compared to conventional injectable [6, 7and^8^, ^9^, ^10^, ^11^- ^12^]. From a pathophysiological standpoint, the results of this study support the concept that TMJ-OA management should extend beyond symptomatic relief to biological modulation of the joint environment. Arthrocentesis addresses the inflammatory burden and mechanical constraints, while PRP targets the underlying degenerative processes by promoting tissue regeneration and inhibiting catabolic pathways. Narrative and systematic reviews have suggested that PRP may exert chondroprotective effects and slow disease progression, thereby offering a disease-modifying potential rather than mere palliation [9, 11]. Despite the favorable outcomes, certain limitations should be acknowledged. The lack of a control group limits direct comparison with other treatment modalities and the relatively small sample size may restrict generalizability. Additionally, variations in PRP preparation protocols across studies remain a challenge, as highlighted in recent systematic reviews of orthobiologic interventions [6, 7 and 10]. Nonetheless, the consistency of statistically significant improvements across all evaluated parameters strengthens the validity of the present findings. The principal limitation of this research was the absence of a control or comparative group, which restricts definitive conclusions regarding the relative superiority of the intervention. Future large-scale randomized controlled trials with standardized PRP preparation protocols and long-term follow-up are warranted to validate and refine treatment predictability. Arthrocentesis with PRP can be confidently integrated into routine clinical practice as an effective minimally invasive management strategy for temporomandibular joint osteoarthritis. In summary, the incorporation of PRP into arthrocentesis protocols resulted in considerable and sustained improvements in pain relief, mandibular function and joint mechanics in patients with TMJ osteoarthritis. The results of this study align closely with existing high-level evidence and further support arthrocentesis combined with PRP as an effective, minimally invasive therapeutic strategy that addresses both the mechanical and biological aspects of TMJ-OA pathophysiology [1, 3, 9, 10].

## Advancement to knowledge:

Osteoarthritis (OA) and internal derangements of the Temporomandibular Joint (TMJ) can be effectively and safely treated with a combination of TMJ arthrocentesis and platelet-rich plasma (PRP) injection. The inclusion of PRP encourages tissue healing, which leads to better long-term results for pain reduction and improved mouth opening, even if arthrocentesis alone offers considerable short-term pain relief by eliminating inflammatory mediators.

## Conclusion:

Arthrocentesis combined with platelet-rich plasma injection established profound and sustained improvement in pain, mandibular function and joint mechanics in patients with temporomandibular joint osteoarthritis. The synergistic integration of mechanical lavage and biologic modulation effectively addressed both inflammatory and degenerative components of the disease. These findings substantiate arthrocentesis with PRP as a potent, minimally invasive and clinically reliable therapeutic modality for TMJ osteoarthritis management.

## Figures and Tables

**Table 1:** Comparison of pain scores (vas) before and after treatment

**Time Interval**	**Mean VAS Score ± SD**	**Mean Difference**	**p value**
Preoperative	7.24 ± 1.02	-	-
1 month post-operative	4.18 ± 0.96	3.06	<0.001*
3 months post-operative	2.87 ± 0.84	4.37	<0.001*
6 months post-operative	1.96 ± 0.73	5.28	<0.001*
*Statistically
significant (p< 0.05)

**Table 2:** Comparison of functional outcomes before and after treatment

**Parameter**	**Preoperative (Mean ± SD)**	**6-Month Follow-up (Mean ± SD)**	**p value**
Maximum mouth opening (mm)	29.6 ± 4.1	41.8 ± 3.6	<0.001*
Presence of joint sounds (%)	71.10%	21.10%	<0.001*
Mandibular function limitation score	3.42 ± 0.62	1.38 ± 0.51	<0.001*
*Statistically significant (p< 0.05)
